# Don’t Be Fooled by Randomness: Valid *p*-Values for Single Molecule Microscopy

**DOI:** 10.3389/fbinf.2022.811053

**Published:** 2022-03-04

**Authors:** Magdalena C. Schneider, Gerhard J. Schütz

**Affiliations:** Institute of Applied Physics, TU Wien, Vienna, Austria

**Keywords:** single molecule microscopy, single molecule localization microscopy, FRET, statistical significance testing, nanoclustering

## Abstract

The human mind shows extraordinary capability at recognizing patterns, while at the same time tending to underestimate the natural scope of random processes. Taken together, this easily misleads researchers in judging whether the observed characteristics of their data are of significance or just the outcome of random effects. One of the best tools to assess whether observed features fall into the scope of pure randomness is statistical significance testing, which quantifies the probability to falsely reject a chosen null hypothesis. The central parameter in this context is the *p*-value, which can be calculated from the recorded data sets. In case of *p*-values smaller than the level of significance, the null hypothesis is rejected, otherwise not. While significance testing has found widespread application in many sciences including the life sciences, it is hardly used in (bio-)physics. We propose here that significance testing provides an important and valid addendum to the toolbox of quantitative (single molecule) biology. It allows to support a quantitative judgement (the hypothesis) about the data set with a probabilistic assessment. In this manuscript we describe ways for obtaining valid *p*-values in two selected applications of single molecule microscopy: (i) Nanoclustering in single molecule localization microscopy. Previously, we developed a method termed 2-CLASTA, which allows to calculate a valid *p*-value for the null hypothesis of an underlying random distribution of molecules of interest while circumventing overcounting issues. Here, we present an extension to this approach, yielding a single overall *p*-value for data pooled from multiple cells or experiments. (ii) Single molecule trajectories. Data from a single molecule trajectory are inherently correlated, thus prohibiting a direct analysis via conventional statistical tools. Here, we introduce a block permutation test, which yields a valid *p*-value for the analysis and comparison of single molecule trajectory data. We exemplify the approach based on FRET trajectories.

## 1 Introduction

One fundamental problem behind the interpretation of biological data relates to the question whether a specific data set agrees with a certain hypothesis or not. Typical examples include the comparative analysis of different subgroups, or the compatibility of data with a specified model. The basic problem arises from the fact that each reproduction of a biological experiment yields a slightly different outcome, irrespective of the quality and precision of the experiment. The reason can be measurement errors or stochastic variations underlying the physical processes. In consequence, it is the interpreter’s problem to judge the compatibility of the data with the hypothesis.

Significance testing has been developed to provide an exact mathematical framework for this problem. The first step is to formulate a null hypothesis, against which the data is to be tested; a typical null hypothesis would be the absence of any effect or difference. Let us introduce as an example the question whether proteins are distributed randomly on a two-dimensional membrane. This question has become central in many fields of cellular biophysics (Garcia-Parajo et al., 2014; Goyette et al., 2019). In our case, the null hypothesis would be a purely random distribution. The idea is now to judge the validity of this hypothesis, based on a binary classifier, which either rejects the hypothesis or not. Naturally, one makes errors in this judgement. One misjudgement is the false rejection of the null hypothesis. The *p*-value together with significance testing is the attempt to quantitatively assess such misjudgements. In a nutshell, the lower the *p*-value, the likelier it is that the data set disagrees with the null hypothesis. Ideally, the researcher defines a significance level *α* before performing the experiment, which is taken as threshold criterion for the decision: any *p*-value below *α* is considered as a rejection of the null hypothesis, whereas any *p*-value greater than *α* would count as agreement.

**FIGURE 1:**
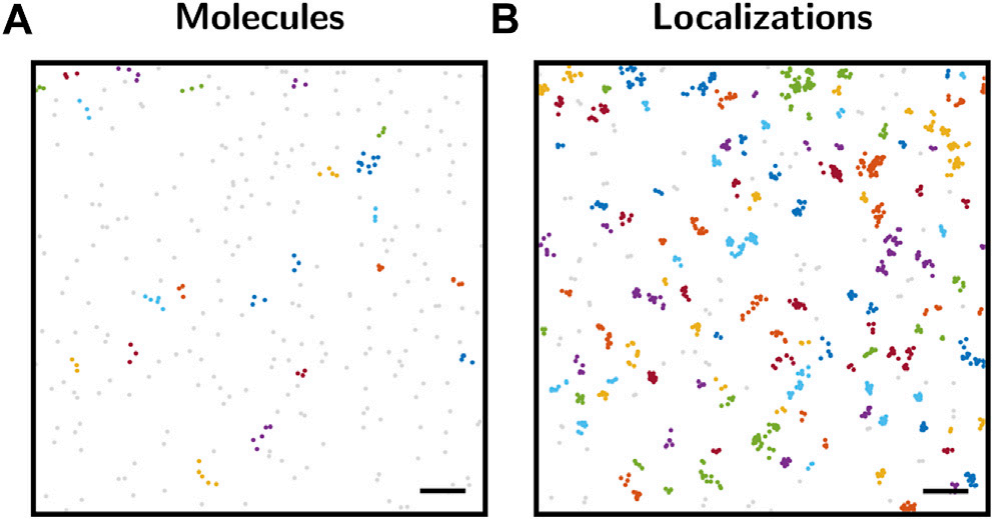
Cluster analysis with DBSCAN. **(A)** Map of molecule positions. Positions were generated by a spatial Poisson point process with a density of 80 points per *μ*m^2^. The point pattern was analyzed by DBSCAN (Ester et al., 1996). Analysis parameters were set to *r* = 50 nm for the search radius and *n* = 3 for the minimum number of points constituting a cluster. The color code represents the cluster assignment. Unclustered points are shown in gray. Although the molecule point pattern represents complete spatial randomness, 22 clusters were identified by DBSCAN. **(B)** SMLM localization map simulated based on the molecule positions from panel **(A)**, including overcounting according to the blinking statistics of SNAP-AF647 (Arnold et al., 2020). The localization map was analyzed by DBSCAN as described in panel **(A)**, yielding 116 clusters. Scale bars: 200 nm.

Significance testing can hence be considered as a powerful tool for a quantitative assessment of a particular experimental outcome. In this context, quantification does not relate to a determination of the magnitude of certain biological parameters, but to a probabilistic assessment of the likelihood of the chosen null hypothesis or the deviation of it. Indeed, as Figure 1A indicates, even random spatial protein distributions contain accumulations that would be picked up as clusters by standard clustering algorithms. Therefore, we consider it important to first globally assess a data set via significance testing before using more detailed analysis tools for a quantification of the biological parameters of interest.

In this manuscript, we provide a guideline how to use *p*-values for the analysis of single molecule microscopy data. In particular, we address the following questions:

• What is the probabilistic basis of the significance level *α* and the *p*-value?
• How can one handle situations in which the distribution of the test statistic under the null hypothesis is not known analytically?
• How can multiple experimental outcomes be combined into one global *p*-value?
• How can one account for correlated data in significance testing?

After a brief introduction into significance testing, we provide the reader of this paper with instructions how to use significance testing in two specific settings:

(i) Detection of protein nanoclusters in membranes. The spatial organization of membrane proteins can be studied in unprecedented detail via single molecule localization microscopy (SMLM). In this superresolution technique, the diffraction limit of light is circumvented by separating the emission of individual fluorophores in time (Sigal et al., 2018; Schermelleh et al., 2019; Lelek et al., 2021; Schütz and Schneider, 2021). After recording and post-processing of thousands of frames, a localization map is obtained. This map is a list of coordinates representing the observed molecule positions. Early studies conducting SMLM experiments on cellular plasma membrane proteins have consistently reported nanoclustering to different degrees (Lillemeier et al., 2010; Rossy et al., 2013; Garcia-Parajo et al., 2014). However, due to blinking of fluorophores the same biomolecule of interest can be detected multiple times during the image acquisition. In combination with localization errors, this leads to localization clusters in the localization maps, which can be easily mistaken for true molecular nanoclustering. Here, we want to address the question of biomolecular nanoclustering in the framework of significance testing.
(ii) Comparative analysis of single molecule trajectories. In SMLM, the high spatial resolution is traded for temporal resolution. To complement this approach, cellular dynamics can be investigated based on the recording of single particle trajectories (Wieser and Schütz, 2008). Similar to SMLM, the density of fluorescent molecules needs to be low enough to distinguish individual molecules. A single molecule is then imaged and tracked over a certain time span, yielding the evolution of a recorded parameter over time. As observed quantity, we considered here the Förster Resonance Energy Transfer (FRET) (Roy et al., 2008). The FRET efficiency corresponds to the non-radiative energy transfer between a donor and acceptor fluorophore, which is directly related to the distance between the two fluorophores. Thus, distances between molecules can be determined at a length scale of a few nanometers. When performing a comparative analysis of recorded samples, one difficulty relates to the correlation within single trajectories. Here, we show how this problem can be solved via a block permutation testing approach.

## 2 Statistical Significance

In the following, we discuss the concept of significance testing for the analysis of biophysical data. As guiding example we will use hypothetical data from SMLM experiments, which shall be analyzed by a 2-color localization microscopy and significance testing approach (2-CLASTA), which we recently developed (Arnold et al., 2020). 2-CLASTA is based on competitive labeling of the same type of biomolecule with labels of two different colors, yielding a 2-color localization map. As the method does not analyze the distribution of localizations directly, but possible correlations between the two color channels, it is independent of the blinking behavior and, in particular, is compatible with any SMLM technique, including PALM, STORM and PAINT [see Supplementary Figure S3 in (Arnold et al., 2020)].

Let us start by considering a pattern generated by a spatial Poisson point process, i.e., complete spatial randomness (Figure 1A), which could correspond to the 2D positions of single protein molecules in a cell membrane. As is apparent from the image, several points will be in close spatial proximity due to random chance alone. This can be easily seen when analyzing the point pattern with clustering methods such as DBSCAN (Ester et al., 1996). Although the point pattern is purely random, multiple clusters were detected by the method.

The situation becomes more severe when considering SMLM-inherent overcounts which arise from repetitive detections due to the blinking kinetics of single dye molecules. Figure 1B shows the same underlying biomolecular distribution as Figure 1A, but now including overcounting which was simulated using typical experimental blinking data. Obviously, more apparent localization clusters arise and are detected by the DBSCAN approach. Thus, a mere analysis of clustering without considering its statistical significance in the context of the global point pattern distribution may yield misleading results.

**FIGURE 2:**
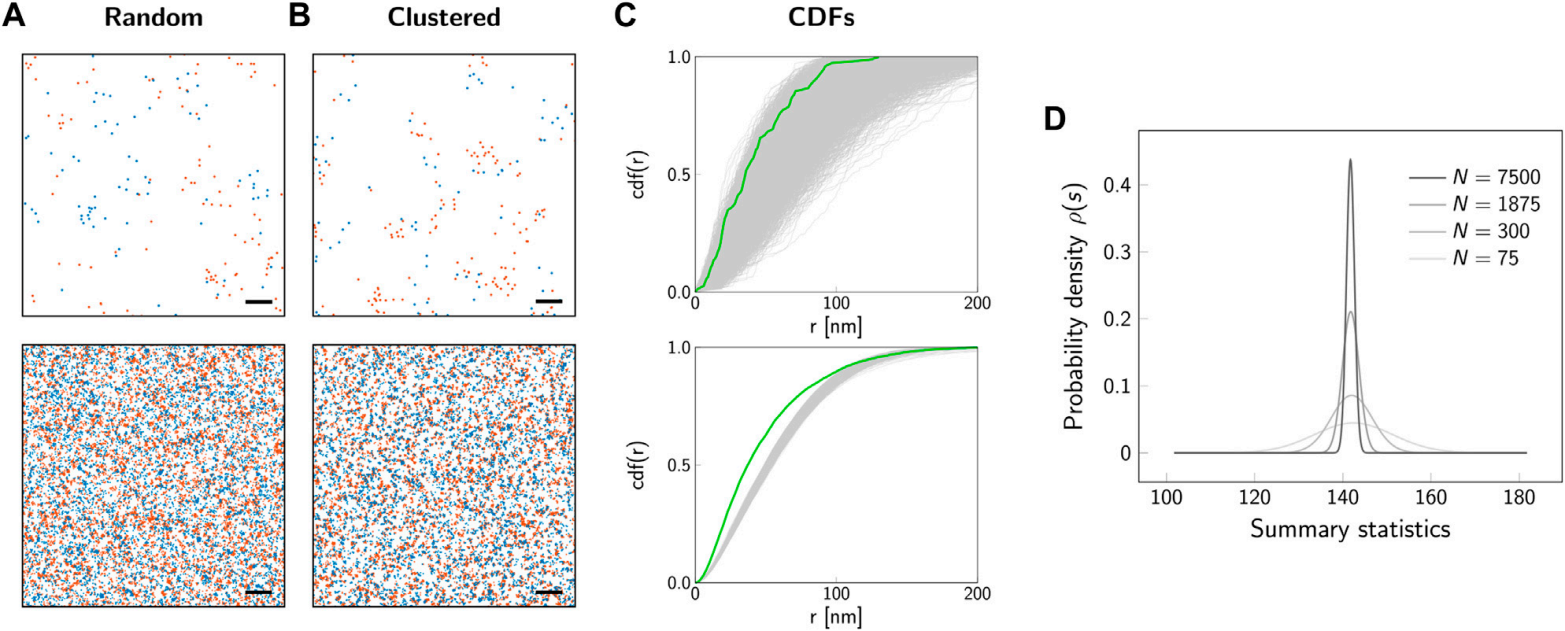
Sampling distribution and influence of sample size. Representative localization maps of an underlying random **(A)** and clustered **(B)** distribution of biomolecules. The simulated regions comprise a number of *N* = 75 underlying molecules (top row) or *N* = 7500 molecules (bottom row). For the clustered scenario, we simulated dimers. The degree of labeling was set to 100*%*. Scale bars: 100 nm (top row), 1 *μ*m (bottom row). **(C)** Cumulative distribution functions (CDFs) of cross-nearest neighbor distances between localizations of the red and blue color channel. Gray lines show the CDFs for 10 000 simulations of random biomolecular distributions. The green line indicates the CDF for the clustered scenario from panel **(B)**. **(D)** Probability distribution of the summary statistic, i.e. the *sampling distribution*. As a summary statistic the integral of the CDF from 0 nm to 200 nm was calculated. The sampling distribution is shown for sample sizes corresponding to a number of molecules from *N* = 75 (light gray line) to *N* = 7500 (black line). The larger the sample size, the narrower is the sampling distribution.

In a statistical analysis, the characteristics of a whole population are estimated based on the analysis of a subsample (Figure 2A); for example, the overall spatial distribution of biomolecules is investigated based on the localization map obtained from a subregion of a cell. The population follows an underlying unknown spatial distribution, which shall be characterized by the statistical test. The *sample* is a data subset which should be representative of the population. For our example of 2-color SMLM data, Figure 2A shows two samples simulated with different sizes of the selected region of interest. The key step now is to identify a sample *summary statistic*, which will be used to infer information about the whole population. In our previous publication, we analyzed the cross-nearest neighbor distances between the two color channels (Arnold et al., 2020). Figure 2C shows the empirical cumulative density function (CDF) for a number of 10 000 different subsamples. In principle, if an analytical and parameterized model of the underlying spatial distribution was available, the empirical CDFs could be fitted and the parameters determined. However, in practice such a model is difficult to establish, making statistical tools a valid choice for approaching this problem. What is apparent at first glance is the rather large spread between the different curves, which is particularly pronounced for smaller subsample sizes. The large spread reflects random effects which lead to variations between subsamples, also if they are drawn from the same underlying population. In the following, we did not use the empirical CDFs directly, but reduced them to the integral over the curve, which was taken as a summary statistics for the subsequent analysis. The sample summary statistics is a random variable that follows a probability distribution (Figure 2D). This probability distribution of the sample summary statistic over all possible random samples of given size *n* is called the *sampling distribution* *ρ*(*s*). The shape of the sampling distribution depends both on the underlying population and the sample size. For increasing sample sizes, the distribution becomes narrower.

**FIGURE 3:**
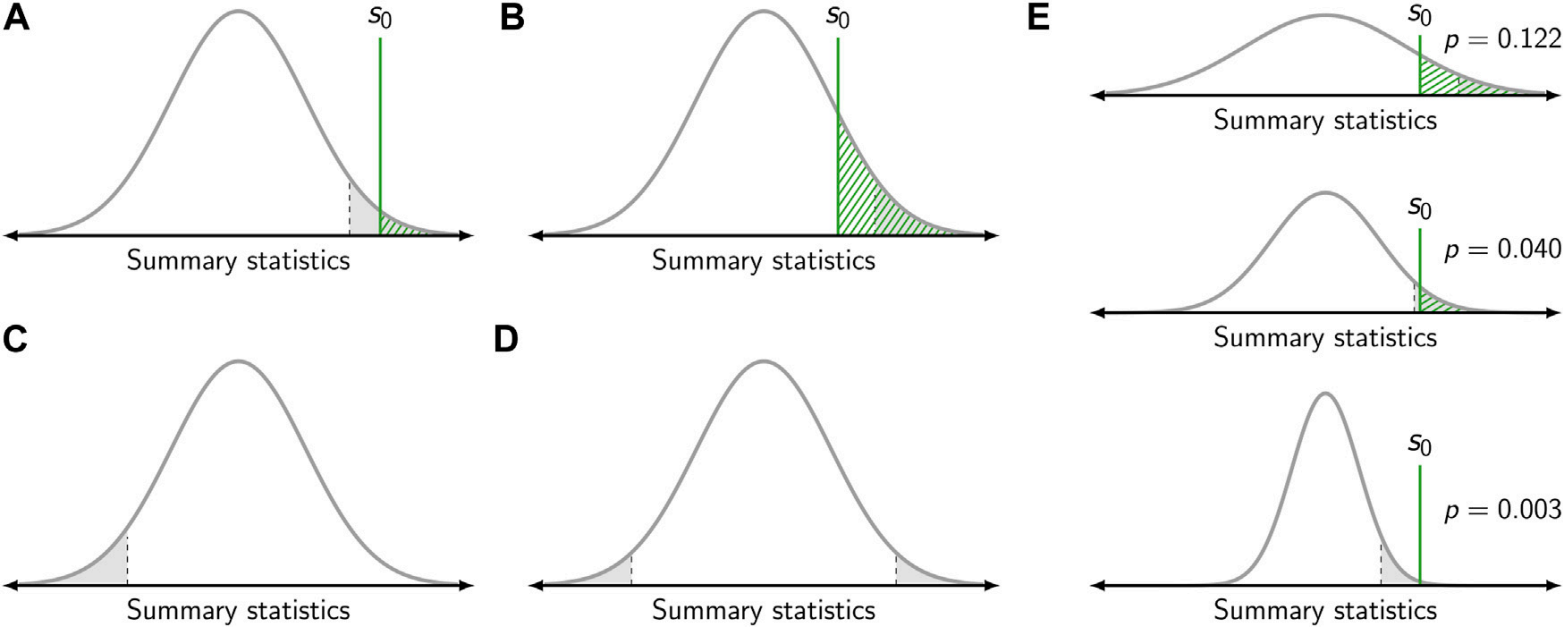
Calculation of the *p*-value. **(A,B)** Gray curves show the sampling distribution of the summary statistic under the null hypothesis. The value of the summary statistic *s*_0_ obtained for one specific sample is shown as green vertical line. The *p*-value corresponds to the dashed green area. The gray area corresponds to the level of significance, which was set to *α* = 0.05. If the *p*-value falls below the level of significance (i.e., *s*_0_ falls within the gray area), the null hypothesis is rejected **(A)**. Otherwise, the null hypothesis is kept **(B)**. In panels **(A)** and **(B)** a right-sided test is depicted. **(C)** Left-sided test. **(D)** Two-sided test. **(E)** Influence of sample size. The same value *s*_0_ of the summary statistics yields a different *p*-value dependent on the sample size (top to bottom). For a small sample size, the sampling distribution is broad and the null hypothesis is kept (top). For a large sample size, the sampling distribution is narrow and the null hypothesis is rejected (bottom).

Let us apply the analysis *via* summary statistics to characterize a simple model, which becomes the basis for the null hypothesis. The null hypothesis *H*_0_ assumes the validity of this model, for example a purely random spatial distribution of all biomolecules. The central idea of significance testing is to quantify the probability for obtaining a certain summary statistics. More precisely, the *p*-value quantifies the probability that drawing from the sampling distribution under the null hypothesis yields a value which is as extreme or more extreme than a given value *s*_0_ (Figure 3A). The *p*-value hence is given by the integral $p=\int_{s_{0}}^{\infty }\rho \left(s\right)ds$ . Typically, *s*_0_ is the value of the summary statistics obtained from an actual experimental observation.

Per definition, the *p*-value is a random variable in the interval [0, 1]. A *p*-value is valid if it fulfills $P\left(p\;\leq \;a\;|\;H_{0}\right)\leq a$ for every 0 ≤ *a* ≤ 1 under the null hypothesis; if equality holds true for all values of *a*, the *p*-value is exact. This definition implies that—under the null hypothesis—the *p*-value shows a uniform distribution. In return, if the *p*-value is not distributed uniformly under the null hypothesis, the null hypothesis does not follow the assumed distribution and thus, the *p*-value is not valid. If the employed test statistic is discrete, the distribution of *p*-values will also be discrete. Hence, the *p*-value cannot be uniformly distributed over the whole interval [0, 1], but can take on discrete values only. Nevertheless, the *p*-value will be distributed uniformly in the sense that $P\left(p\leq a\;|\;H_{0}\right)=a$ , if *a* is a value that can be taken on by the *p*-value, and $P\left(p\leq a\;|\;H_{0}\right)<a$ otherwise. Hence, the *p*-value is valid.

As the *p*-value is based on the sampling distribution, it not only depends on the population but also the sample size (Figure 3E). Hence, the same outcome for a summary statistics may yield different *p*-values dependent on the sample size as the width of the sampling distribution varies.

The *p*-value allows to assess statistical significance, i.e., whether a result for a test statistic is more extreme than what can be expected from random chance. It describes how incompatible the observed data are with the statistical model specified by the null hypothesis. Thus, the *p*-value can be used to conduct a hypothesis test, in which the null hypothesis *H*_0_ is tested against the alternative hypothesis *H*_1_. Of note, the two hypotheses *H*_0_ and *H*_1_ should be mutually exclusive and their union should cover the whole range of possible outcomes. The test decision, i.e., whether the null hypothesis is rejected or kept, is based on the *p*-value and a chosen threshold termed the level of significance *α*. The null hypothesis is rejected if the obtained *p*-value is lower than or equal to *α*. If the *p*-value is larger than *α*, the null hypothesis is kept (Figure 3).

Let us consider three different scenarios for the application of significance testing to the analysis of SMLM data. First, a test shall be performed for the null hypothesis of a random distribution of biomolecules against the alternative hypothesis of (nano-)clustering. As indicated in Figures 2B,C, spatial clustering leads to a steeper increase in the empirical CDFs concomitant with a higher value of the determined summary statistics *s*_0_. In this case, it is hence recommendable to use the right-sided *p*-value (Figure 3A) and only reject the null hypothesis in case of extremely high values of *s*_0_. Second, we assume as alternative hypothesis a repulsion of the molecular positions. In this case, molecules would be dispersed across the field of view, concomitant with a smaller value of the determined summary statistics *s*_0_. Consequentially, the left-sided *p*-value would be used for the test (Figure 3C). Third, it may be the case that any deviation from a random distribution is of interest to the experimentalist. In this case, one would opt for choosing the two-sided *p*-value, and reject the null hypothesis both in case of extremely high and low values of *s*_0_ (Figure 3D).

For a valid analysis, the value of the significance level *α* needs to be specified *a priori*, i.e., before calculating the *p*-value for a particular experiment (Shine, 1980). Only in this case the level of significance corresponds to the false positive rate of the test. If the level of significance is selected *a posteriori*, the researcher may be biased in the choice of *α* dependent on the obtained *p*-value. Thus, the probability for an incorrect rejection of the null hypothesis will be affected.

For the interpretation of results it should be kept in mind that the outcome of a test decision, i.e. the rejection or acceptance of the null hypothesis, may be incorrect. The type I and type II error quantify the probability of a false decision. The *type I error* corresponds to false positives: The null hypothesis is erroneously rejected, i.e. an observed effect is assumed to be real although it is due to random chance alone. Interestingly, the probability of a type I error—i.e. the false positive rate—is directly determined by the chosen level of significance. For a valid *p*-value it holds that $P\left(p\leq \alpha \;|\;H_{0}\right)\leq \alpha$ for all *α* ∈ [0, 1]. In other words, the probability of falsely rejecting the null hypothesis is smaller than or equal to *α*. For an exact *p*-value the false positive rate is exactly *α*. A type II error occurs in case of false negatives: the null hypothesis is kept, although the alternative hypothesis is true. Of note, the probability of a type II error depends on the sample size; with increasing sample size the sampling variation decreases and even small differences in the summary statistics can be attributed to truly existing effects instead of random noise.

**FIGURE 4:**
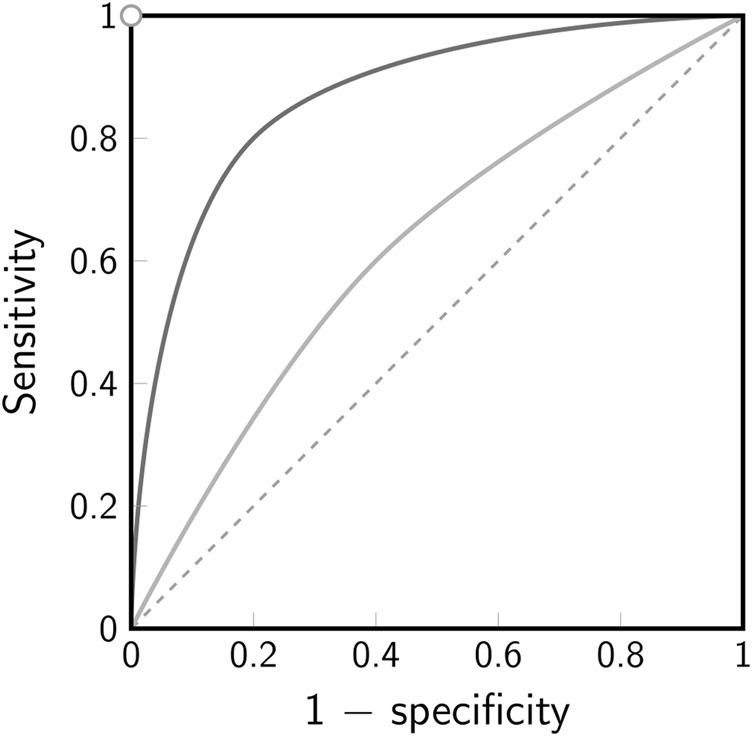
Receiver operating characteristic (ROC). The ROC curve illustrates the trade-off between sensitivity and specificity for a binary classifier. The true positive rate (sensitivity) is plotted against the false positive rate (1-specificity). Note that for a hypothesis test the false positive rate corresponds to the chosen level of significance *α*. The white dot in the top left corner indicates the point of perfect discrimination, the dashed line indicates the line of no discrimination. The solid lines indicate two scenarios for a binary classifier with low discrimination (light gray) and better discrimination (dark gray).

The outcome of the test decision always depends on the chosen level of significance *α*, which usually affects the probabilities for a type I and type II error. Notably, lowering the chance for one error increases the other. The trade-off between the two errors is best visualized by a ROC (receiver operating characteristic) curve (Figure 4). In a ROC curve, the true positive rate (= 1 − false negative rate = sensitivity) of a test is plotted against the false positive rate (= 1 − true negative rate = 1 − specificity). A perfect binary classifier would yield a point in the top left corner (0, 1) of the ROC plot, corresponding to 100*%* sensitivity and 100*%* specificity. In general, however, a certain probability for either of the two types of errors in the classification remains. A classifier based on random guesses would yield a ROC curve given by the diagonal (*line of no discrimination*, indicated by the dashed line in Figure 4).

## 3 2-CLASTA

**FIGURE 5:**
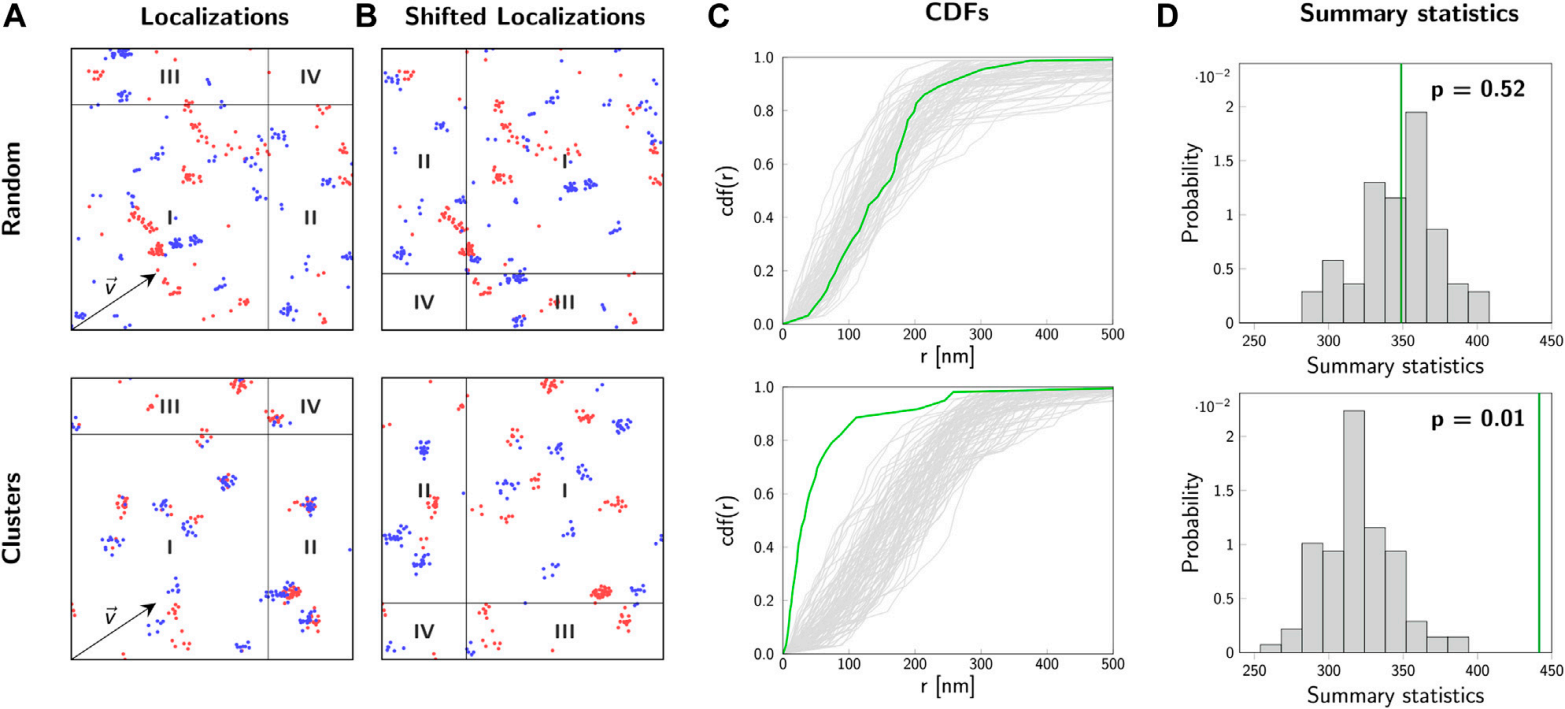
2-CLASTA method. Analysis of localization maps with 2-CLASTA. **(A)** Simulated two-color localization maps for a random (top) and a clustered (bottom) distribution of biomolecules. Images show a 2 × 2 *μ*m^2^ region. For the simulation of blinking we used experimental data obtained for SNAP-AF488 (blue channel) and SNAP-AF647 (red channel). **(B)** Shifting all localizations of the blue color channel by the shift vector $\vec{v}$ breaks correlations between the two color channels. **(C)** The CDF of cross-nearest neighbor distances, *r*, between the two color channels is plotted in green for the localization data shown in panel **(A)**. The functions cdf_rand_(*r*) of *n* = 99 control curves, generated with randomly chosen toroidal shifts, are depicted in light gray. **(D)** As a summary statistics, the integral of the CDFs was calculated. Based on the rank of the summary statistics *s*_0_ for the original data (green line), we calculated a *p*-value *p* = 0.52 for the random case, and *p* = 0.01 for the clustered case. Panels **(A–C)** adapted from (Arnold et al., 2020), CC BY 4.0 (https://creativecommons.org/licenses/by/4.0/).

Often, the sampling distribution of the summary statistics under the null hypothesis is not known analytically. In our 2-CLASTA method, we create estimations of the summary statistics under the null hypothesis of a random biomolecular distribution directly from the recorded localization maps. For this, a toroidal shift is applied to one of the color channels (Figures 5A,B): All localizations are shifted by a random vector $\vec{v}$ and moved back into the regions of interest according to periodic boundary conditions. The toroidal shift breaks possible correlations between the two color channels while conserving the characteristics of the localization map of each individual channel. By repeating this procedure for randomly chosen shift vectors, a set of random control images can be generated on the computer which allows to calculate the corresponding CDFs of cross-nearest neighbor distances (Figure 5C). Each integral of these CDFs gives an estimate of the summary statistics. Typically, we calculated *n* = 99 toroidal shifts, yielding a good approximation of the sampling distribution of our summary statistics (Figure 5D).

**FIGURE 6:**
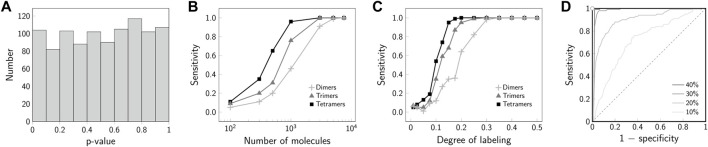
Specificity and sensitivity of 2-CLASTA. **(A)** Specificity. The histogram of *p*-values shows a uniform distribution under the null hypothesis of a random biomolecular distribution. **(B)** Sensitivity for a varying number of observed molecules. Results are shown for simulations of dimers (**+**), trimers (▲) and tetramers (■). The sensitivity increases with a increasing number of observed molecules. **(C)** Sensitivity for varying degree of labeling. Results are shown for simulations of dimers (**+**), trimers (▲) and tetramers (■). For a degree of labeling above 30*%*, maximum sensitivity can be achieved. **(D)** ROC curves are plotted for simulations of dimers with a degree of labeling varying from 10*%* (light gray) to 40*%* (black). With increasing degree of labeling the discrimination power of the test increases, i.e., the ROC curve approaches the point of perfect classification in the top left corner. The gray dashed line indicates the line of no discrimination. The sensitivity for each parameter set was determined based on 100 independent simulations. Panels **(A–C)** adapted from (Arnold et al., 2020), CC BY 4.0 (https://creativecommons.org/licenses/by/4.0/).

Finally, the obtained value *s*_0_ of the summary statistics for the original data is compared with the values *s*_*i*_ obtained for the sampling distribution under the null hypothesis. For the calculation of a *p*-value, all values of the set $S≔\left\{s_{i}\;|\;i=0,\ldots ,n\right\}$ are sorted in descending order and a rank is assigned to each value according to its position in the ordered sequence: A value *s*_*i*_ has the rank *j* if it is the *j*th largest element; consequently, the largest value of the set has rank 1. Since we want to test the null hypothesis of a random distribution against the alternative hypothesis of nanoclustering, we are interested whether the original data shows a tendency towards shorter nearest neighbor distances. Deviations towards larger distances are not considered of importance here. Hence, we calculate

$$p=\frac{rank\left(s_{0},S\right)}{n+1},$$ (1)

where $rank\left(s_{0},S\right)$ denotes the descending rank of *s*_0_ within the set S . Under the null hypothesis the calculated values *p* show the expected uniform distribution in the interval [0, 1] (Figure 6A) and, hence, can be interpreted as right-sided *p*-values.

In order to perform the significance testing it is important now to select a significance level *α*. In the following, we chose *α* = 0.05, equivalent to a 5*%* false rejection rate of the null hypothesis of a random biomolecular distribution. In our approach, the obtainable *p*-values are constraint to discrete values in the set $\left\{\frac{i}{n+1}\;|\;i=1,\ldots ,n+1\right\}$ . It is hence important to ensure that $\alpha >\frac{1}{n+1}$ . In the example shown in Figure 5, we obtained a *p*-value of 0.52 for the random biomolecular distribution (top row) and a *p*-value of 0.01 for the clustered scenario (bottom row). Our test hence yielded a correct test decision in both cases.

While the validity of a *p*-value is described by the correct rejection rate of the null hypothesis, its power is linked to the sensitivity for detecting aberrant scenarios. In the following, we give examples of the sensitivity of 2-CLASTA to detect biomolecular oligomers from dimers up to tetramers. As expected, the sensitivity strongly depends on the number of analyzed molecules within the region of interest (Figure 6B). This reflects the larger spread of the sampling distribution for small data sets (cf. Figure 2). For data sets containing more than 3000 molecules, the identification even of dimers works robustly with a sensitivity above 80*%*. In a biological experiment, it is difficult to achieve a degree of labeling of 100*%*. With decreasing degree of labeling, two-color colocalization will be out-diluted by apparent monomeric signals, which arise from underlabeled oligomers. Figure 6C shows that a degree of labeling of 30*%* is sufficient to reliable detect all analyzed cluster scenarios. The improved sensitivity for high degree of labeling is also apparent in the ROC plot (Figure 6D). While for 10*%* degree of labeling we obtained results close to the line of no discrimination, 40*%* degree of labeling approaches the point of perfect discrimination extremely well. Of note, we showed previously that minor chromatic aberrations hardly affect the analysis, as they do not break correlations between the two color channels [see Supplementary Figure S4 in (Arnold et al., 2020)]. If one desires to use a single dye only (e.g. due to its superior photophysical properties), one can perform an Exchange-PAINT experiment (Jungmann et al., 2014) with two different docking strands, which would then be taken as the two different channels in the 2-CLASTA analysis.

**FIGURE 7:**
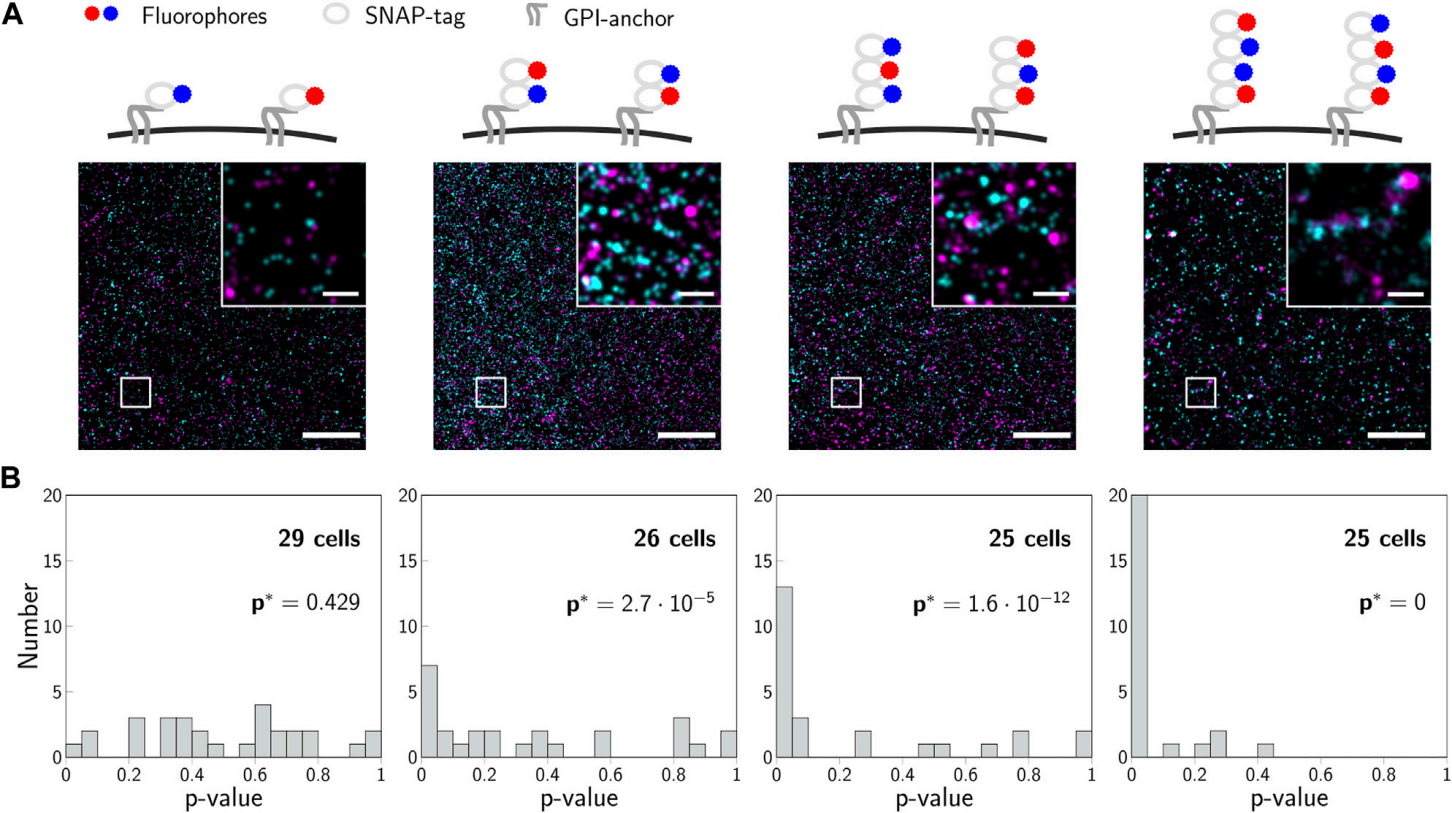
Experimental validation of 2-CLASTA. **(A)** Illustrations and representative localization maps recorded for SNAP-monomers, -dimers, -trimers and -tetramers (left to right). Scale bars: 250 nm (inset) and 2 *μ*m. **(B)** Histograms of *p*-values obtained for multiple recorded cells, which were analyzed individually with the 2-CLASTA method. For each SNAP construct, we also calculated the joint *p*-value p* according to Eq. 2, with the threshold *p*_0_ = 0.05. Panels **(B)** and **(C)** adapted from (Arnold et al., 2020), CC BY 4.0 (https://creativecommons.org/licenses/by/4.0/).

For a validation experiment, we previously generated concatamers of SNAP-tags fused to a GPI-anchor, which are located in the cellular plasma membrane (Arnold et al., 2020). The fusion-constructs were labeled with mixtures of blue and red substrates so that similar degrees of labeling were achieved for both colors (Figure 7A). For each construct, we recorded 2-color SMLM experiments on at least 25 cells, analyzed them according to the 2-CLASTA method and determined a *p*-value for each image (Figure 7B). The resulting histograms in the case of monomeric constructs yielded a rather uniform distribution, whereas all other constructs showed a substantial deviation from this uniform distribution, with an increased fraction of small *p*-values with increasing oligomer degree. Importantly, the rather small region of interest and suboptimal degree of labeling generally compromise sensitive identification of the presence of oligomers from a single experiment, yielding multiple experiments with an outcome above the significance threshold.

## 4 Accounting for Multiple Experiments

In order to assess the overall outcome of multiple experiments in a single joint statistical analysis, one straightforward option seems to be taking the minimum of all observed *p*-values as overall *p*-value and reject the null hypothesis if this minimum *p*-value is significant. However, this procedure is not valid as it drastically increases type I errors, i.e. the false positive rate. This can be seen from a simple example: For *m* independent experiments, the probability that none of the obtained *p*-values is significant under the null hypothesis can be calculated as (1 − *α*)^*m*^. For *m* = 10 experiments and a significance level of *α* = 0.05, the probability to incorrectly obtain a significant result would be 1 − (1 − 0.05)^10^ = 0.4, which is much higher than the significance level. This is also evident from the probability distribution of the minimum *p*-value. In case of a continuous *p*-value, the distribution of the minimum of *m* uniformly distributed values *p*_min_ =  min(*p*_1_,…,*p*_*m*_) is not uniform but follows the probability density function $m{\left(1-p_{\text{min}}\right)}^{m-1}$ .

In order to adjust the overall *p*-value for *m* experiments, Wieser et al. (Wieser et al., 2008) suggested to apply the transformation function $p^{*}=1-{\left(1-p_{\text{min}}\right)}^{m}$ , yielding a uniform distribution of p* on the interval [0, 1]. However, in case of bootstrapping and Monte Carlo approaches the *p*-value is not continuously distributed, but can only take on discrete values $\frac{i}{n+1}$ for *i* = 1, …, *n* + 1, where *n* is the number of bootstrapped or simulated control samples. Therefore, the lower bound of p* is $1-{\left(\frac{n}{n+1}\right)}^{m}$ , which approaches 1 for *m* approaching infinity. Hence, for a large number of experiments *m* the null hypothesis would never be rejected.

In case of discrete *p*-values we propose here a different method to adjust the *p*-value for multiple experiments. The *p*-values for single experiments then show a discrete uniform distribution under the null hypothesis given by $P\left(p=\frac{i}{n+1}\right)=\frac{1}{n+1}$ for *i* = 1, …, *n* + 1, where *n* is the number of simulated controls. In order to combine the *p*-values obtained from multiple experiments, we can determine whether the number of observed *p*-values below a user-defined threshold *p*_0_ agrees with a discrete uniform distribution. Under the null hypothesis, the probability to obtain a *p*-value below or equal to the threshold *p*_0_ in exactly *k* out of *m* experiments is given by a Binomial distribution *B*(*k* | *p*_0_, *m*).

Therefore, we can perform a Binomial test in order to determine whether the *p*-values obtained from *m* independent experiments agree with the null hypothesis. In general, one is interested in identifying significant results characterized by a high proportion of low *p*-values. Hence, a right-sided Binomial test of the null hypothesis is appropriate. The overall *p*-value p* for multiple experiments is calculated as

$$p^{*}=P\left(X\geq k\right)=\overset{m}{\underset{i=k}{\sum }}B\left(i\;|\;p_{0},m\right)=\overset{m}{\underset{i=k}{\sum }}\left(\begin{array}{c} m\\ i \end{array}\right)p_{0}^{i}{\left(1-p_{0}\right)}^{m-i}=1-\overset{k-1}{\underset{i=0}{\sum }}\left(\begin{array}{c} m\\ i \end{array}\right)p_{0}^{i}{\left(1-p_{0}\right)}^{m-i},$$ (2)

where *k* is the number of observed *p*-values below the chosen threshold *p*_0_, and *m* the number of performed experiments. If p* is smaller than the chosen level of significance α* for the joint analysis of experiments, the null hypothesis is rejected.

**FIGURE 8:**
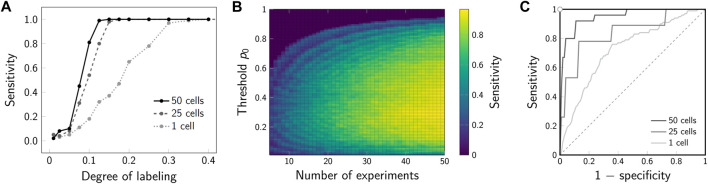
Sensitivity for joint analysis of multiple experiments. **(A)** Sensitivity for varying degree of labeling. Dimers were simulated and analyzed with 2-CLASTA. The sensitivity for analysis of a single experiment is shown by the dotted line. The sensitivity in case of joint analysis of 25 and 50 cells is shown by the dashed and solid lines, respectively; a threshold of *p*_0_ = 0.05 was chosen. **(B)** Influence of the threshold *p*_0_ and the number of experiments on the sensitivity. The obtained sensitivity is indicated by color. **(C)** ROC plot for joint analysis of multiple experiments. For the simulations in panels B and D, the degree of labeling was set to 10*%*. Sensitivity was calculated from 100 simulation runs; the level of significance was set to α* = 0.05.

The increase in sensitivity for the joint analysis of multiple 2-CLASTA analyses compared to a single experiment is shown in Figure 8A. For this, dimers were simulated with varying labeling efficiency, assuming a 1 : 1 label ratio between the two colors. A joint analysis of 25 or 50 simulated experiments yielded a drastic increase of the sensitivity compared to the analysis of a single experiment only. Interestingly, the method is very robust with regard to the chosen threshold *p*_0_ (Figure 8B). As expected, the higher the number of analyzed experiments, the higher is the sensitivity of the method. Also in the ROC plot we observed a strongly improved performance that approaches the ideal test (Figure 8C). The proposed joint analysis of all performed experiments was also applied to the experimental results obtained on the SNAP constructs from our previous paper (Arnold et al., 2020). The calculated overall *p*-values for multiple experiments are indicated as p* in Figure 7B. As anticipated, the null hypothesis of a random protein distribution was not rejected for the monomeric 1-SNAP construct. For all the oligomeric constructs representing dimers, trimers and tetramers, the null hypothesis was rejected and the biomolecular distribution was correctly identified as clustered.

## 5 Single Particle Trajectories

As a final example, we will discuss here the application of significance tests to the analysis of single particle trajectories. In practice, such trajectories suffer from a limited observation time due to restrictions in the overall imaging experiments, diffusion of the molecule out of the region of interest, or photobleaching of the fluorescence marker molecules. In the following, we present a guideline how to compare sets of single particle trajectories recorded under two different conditions *A* and *B* via permutation tests (Good, 2000).

**FIGURE 9:**
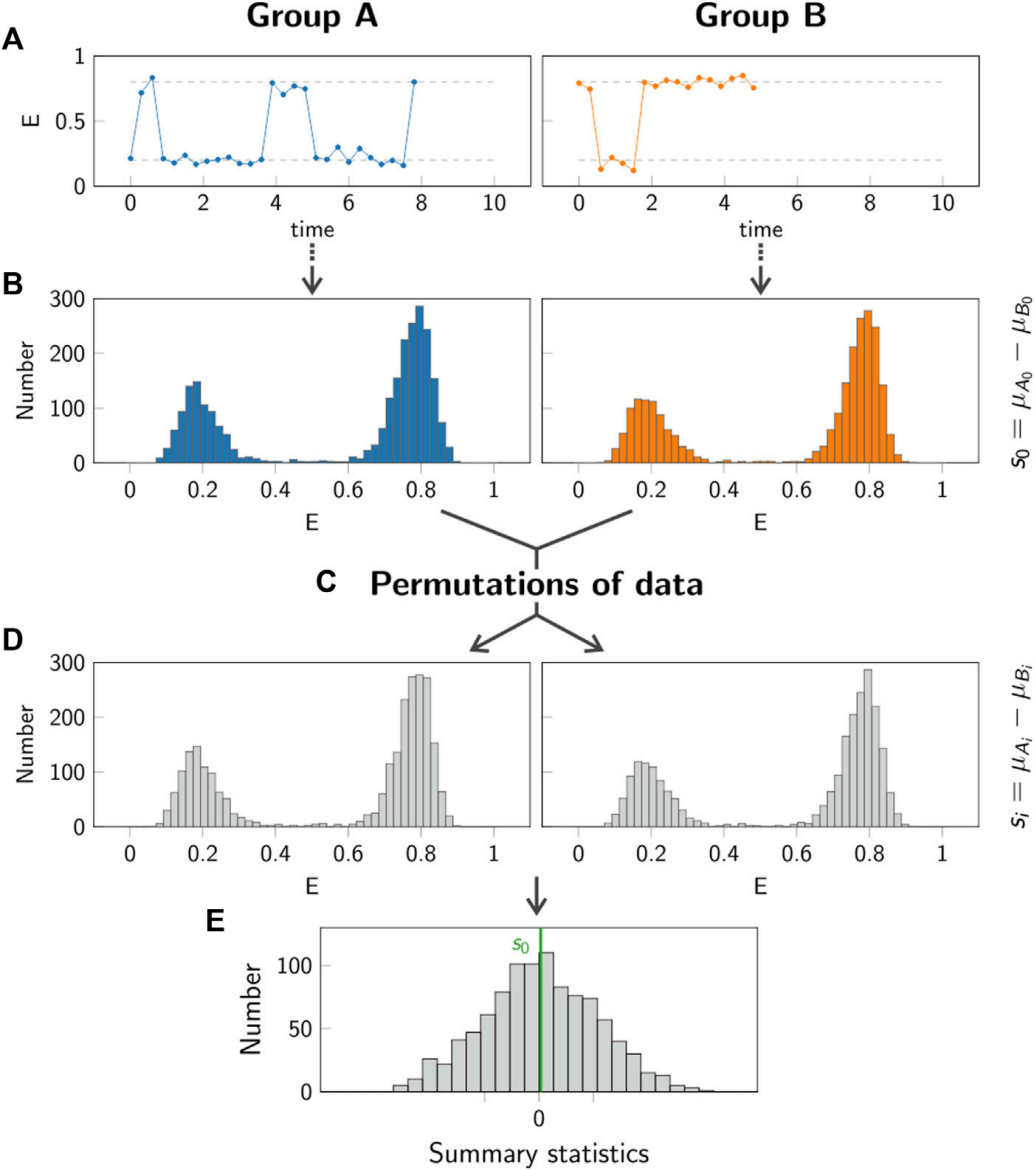
Principle of permutation test. In the permutation test, the group *A* (left column, blue) is compared to the group *B* (right column, orange). Step **(A)**: Individual single molecule trajectories are recorded for both groups *A* and *B*. Step **(B)**: The data obtained from all trajectories in each group is pooled. The summary statistics *s*_0_ is calculated in order to compare the two groups. Step **(C,D)**: Random permutations of the data in the two groups are generated, yielding new samples *A*_*i*_ and *B*_*i*_. For each permutation, the summary statistics *s*_*i*_ is calculated. Step **(E)**: The *p*-value is obtained by comparing the value *s*_0_ (green line) to the values *s*_*i*_ obtained for 1000 permutations.

Figure 9 shows the typical workflow of such a test approach. All data points recorded under condition *A* and *B* are combined, yielding the average values *μ*_*A*_ for sample *A* and *μ*_*B*_ for sample *B*, respectively. We choose here as summary statistics the difference *s*_0_ = *μ*_*A*_ − *μ*_*B*_. One may use as a realization of the null hypothesis, i.e., no difference between the sample *A* and *B*, a random splitting of the combined data sets in two new subsamples *A*_*i*_ and *B*_*i*_ each containing the same amount of data points as the original samples *A* and *B*. For each permutation, a new sample statistics $s_{i}=\mu_{A_{i}}-\mu_{B_{i}}$ is calculated. Finally, *s*_0_ is compared with the sampling distribution of all obtained values of *s*_*i*_. The *p*-value is obtained via the rank of *s*_0_ as described in Section 3, Eq. 1.

To evaluate this approach, we simulated representative single molecule trajectories, consisting of a time series of a recorded parameter *E*(*t*). This could be the FRET efficiency in a single molecule FRET experiment, the size of displacement steps in a single particle tracking experiment, the excited state lifetime in a spectroscopic experiment, to name a few. Representative trajectories for this evaluation are shown in Figure 9A.

**FIGURE 10:**
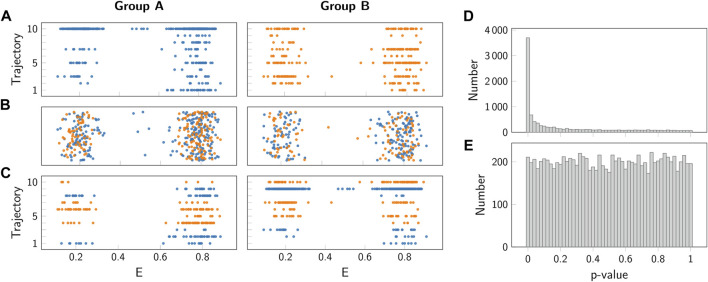
Comparison of permutation test and block permutation test. **(A)** Representative simulated FRET efficiencies for 10 simulated trajectories of group *A* (left, blue) and *B* (right, orange). Both groups were simulated using the same parameters (see Section 7). **(B)** Representative random permutation of the data from panel **(A)**. **(C)** Representative block permutation of the data from panel **(A)**. Here, data from individual trajectories is kept together. **(D,E)** Histogram of *p*-values obtained for the standard permutation test panel **(D)** and the block permutation test panel **(E)**. As a test summary statistics the difference between the means of the groups *A* and *B* was taken. As both groups were simulated using the same parameters, the null hypothesis of no difference between the groups was fulfilled. Importantly, only the block permutation test provides a uniform distribution and hence a valid *p*-value.

To verify the validity of this approach for calculating a correct *p*-value, we plotted the distribution of obtained *p*-values under the null hypothesis for thousand repetitions of this hypothetical experiment. As discussed in Figure 6A, a valid *p*-value has to show a uniform distribution under the null hypothesis. Interestingly, in our case we observed massive deviations from such a uniform distribution, with a strong peak for small *p*-values (Figure 10D). When applied to a significance test, the experimenter would hence incorrectly reject the null hypothesis too often.

To understand the reason for this incorrect judgement, let us have a closer look on the single molecule trajectories. In our case, we did not assign binary values to a time series randomly, but instead we considered states with a specific duration characterized by transition rate constants. In consequence, the data used for the sampling distribution are not independent, thereby violating a basic assumptions of most significance testing approaches.

To solve this problem, we used a block permutation test approach. In this approach, the trajectories recorded in the samples *A* and *B* are not split, but instead kept together for the permutations performed in step *C* in Figure 9. This approach correctly accounts for the correlations in the trajectories when generating the permuted samples *A*_*i*_ and *B*_*i*_. Indeed, application of this approach leads to uniform distribution of the *p*-value under the null hypothesis (Figure 10E).

**FIGURE 11:**
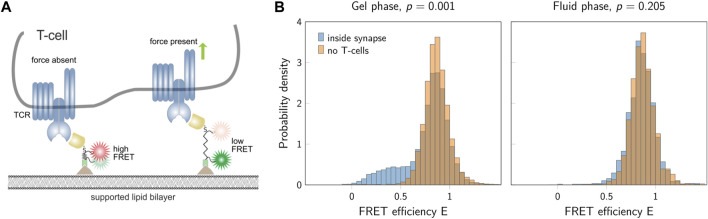
FRET data from T-cell experiments. **(A)** Sketch of a T-cell and the force sensor. If no force is applied, the sensor shows a high FRET signal (left). In the presence of forces, a low FRET signal is detected (right). **(B)** FRET efficiencies for a gel-phase bilayer (left) and a fluid-phase bilayer (right). The two histograms show data inside the T-cell synapse (blue) and in the absence of T-cells (orange). The *p*-values indicated in the figure are the results of the block permutation test. The standard permutation test would yield a *p*-value of 0.001 and 0.004 for the gel- and fluid-phase bilayer, respectively. Figure adapted from (Göhring et al., 2021), CC BY 4.0 (https://creativecommons.org/licenses/by/4.0/).

We applied the new method to experimental data recorded previously in our lab, which shows the analysis of single molecule FRET trajectories of a molecular force sensor (Göhring et al., 2021). Briefly, T-cells were seeded on a glass supported lipid bilayer, which was functionalized by specific proteins to activate the T-cells. One of these proteins was a force sensor, carrying a spider silk spring element, which connected a membrane anchoring motive with a specific ligand to the T-cell receptor on the T-cell surface. A donor and an acceptor fluorophore were conjugated to the spider silk region and used for reading out the elongation of this spring element via single molecule FRET, which was eventually used to calculate forces. For each experimental run, two different conditions were recorded: Condition *A* (orange) corresponding to the FRET signal of the force sensor without T-cells, and condition *B* (blue) corresponding to the FRET signal recorded in the synapse between the T-cell and the supported lipid bilayer (Figure 11).

When using a gel-phase lipid bilayer, we observed a clear difference between the two conditions corroborated by a highly significant *p*-value. In contrast, fluid-phase bilayers yielded similar results when comparing the two conditions. The block permutation test yielded *p* = 0.205, and hence, no significant deviation from the null hypothesis for a chosen significance level of *α* = 0.01. Of note, the simple permutation test not accounting for correlations would have yielded a *p*-value of *p* = 0.004, and hence, would have indicated a significant difference between the two samples.

## 6 Discussion

Calculating a *p*-value can be very useful for researchers in order not to be fooled by random chance. Random variations often lead to outcomes that can easily be misinterpreted as interesting patterns. The *p*-value allows to quantitatively assess whether an observed effect likely occurred due to random chance alone or whether it is worth to study the effect in more detail. As an example, SMLM experiments on cellular proteins often revealed notable deviations from a random distribution of localizations (Lillemeier et al., 2010; Rossy et al., 2013). When analyzed via cluster detection methods such as Ripley’s K function, DBSCAN or modified versions of it (Ripley, 1977; Ester et al., 1996; Rubin-Delanchy et al., 2015), one would arrive at the conclusion of biomolecular clustering in the sample of interest. More elaborate analysis allowed to include the aspect of overcounting due to the inherent blinking processes in SMLM (Annibale et al., 2011; Sengupta et al., 2011; Baumgart et al., 2016; Rossboth et al., 2018; Bohrer et al., 2021), putting some of these clusters into question. But also the application of these refined methods is not straight forward as it requires either the adjustment of user-defined parameters, or the recording of single molecule blinking traces.

In this paper, we proposed a different approach towards such problems. Our idea is not a direct quantitative interpretation of the data, but a statistical assessment of hypotheses (Baddeley and Bewersdorf, 2018). If one opts for such an approach, two issues need to be considered:

(i) Which hypothesis describes the problem most appropriately? In an SMLM experiment a typical example for the null hypothesis would be: *The spatial distribution of detected localizations agrees with a random point pattern*. Due to overcounting this hypothesis will likely be rejected in most data sets. A modified hypothesis may thus be: *The spatial biomolecular distribution agrees with a random point pattern.* We addressed this hypothesis in Section 3 of this paper. If also this hypothesis is rejected, one may opt for coming up with more precise hypotheses about the lateral extension of the biomolecular clusters and the degree of clustering. The result of such an approach will hence be rather similar to the classical quantitative approaches; its advantage is that it additionally provides a *p*-value. We previously used such a strategy to test experimental results against thousands of quantitatively well-defined hypotheses to analyze single molecule tracking data (Wieser et al., 2008; Axmann et al., 2012) and FRET recordings (Schrangl et al., 2018).
(ii) How can we derive a *p*-value to test the null hypothesis? Here, the major limitation comes from the fact that the underlying sampling distribution of the summary statistics is typically unknown. In principle, one could derive such a sampling distribution analytically or generate it on the computer. The drawback of it is that additional experiments are required to obtain the molecular parameters describing the behavior of the single fluorophores (Platzer et al., 2020). We opted here for a different approach, which makes use of the experimental data themselves: In case of 2-CLASTA (Section 3), a toroidal shift was used for reassigning molecular positions in one of the two color channels, which allowed to calculate a set of computer-generated control samples representing the null hypothesis of the absence of correlations between the two color channels. In case of the single particle tracking experiments (Section 5), the problem was different: Now, correlations present in the data had to be correctly accounted for also in the computer-generated control samples. We achieved this using a block permutation strategy.

It should be noted that the *p*-value and significance testing have recently become an issue of dispute. A variety of articles and comments have been published, both arguing for and against the validity of *p*-values (Halsey et al., 2015; Lazzeroni et al., 2016; Altman and Krzywinski, 2017; Amrhein et al., 2019; Lakens, 2021). This is mainly due to misinterpretations of how to correctly interpret *p*-values. In 2016, the American Statistical Association released a statement addressing several misconceptions about the *p*-value (Wasserstein and Lazar, 2016).

Importantly, the *p*-value is not the probability that the null hypothesis is true, but rather indicates how compatible the observed data are with the null hypothesis. In other words, a rejection of the null hypothesis does not prove that the null hypothesis is false: The null hypothesis could still be true, but instead a very unlikely event occurred. Vice versa, not rejecting the null hypothesis does not prove its truth. Strictly speaking, a non-significant test result has no relevance at all.

The test decision always depends on the chosen level of significance, which usually affects the probabilities for a type I and type II error. Notably, lowering the chance for one error increases the other, and a certain probability for either error always remains. Hence, an outcome of a test should never be taken as a proof for *proving* a hypothesis.

One major issue is known as *fishing for *p*-values*. In case of a true null hypothesis, there is still a certain probability to obtain a significant *p*-value. For one single hypothesis test, this probability corresponds to the level of significance *α*. If one conducts multiple experiments and performs a hypothesis test for each, the probability to obtain a significant *p*-value is given by 1 − (1 − *α*)^*m*^, where *m* denotes the number of experiments. Evidently, 1 − (1 − *α*)^*m*^ approaches 1 for large values of *m*, i.e. for a large number of experiments, one will obtain by chance a significant *p*-value with high probability.

As *p*-values have been controversial, the use of alternatives such as estimation statistics and confidence intervals have been encouraged (Claridge-Chang and Assam, 2016). A confidence interval is an interval estimate for an unknown parameter. It is always associated with a certain confidence level, which corresponds to the percentage of confidence intervals containing the true parameter. Nevertheless, both *p*-values and confidence intervals are based on the same statistical theories. Inferences about statistical significance based on either are directly linked: If a *p*-value is smaller than the level of significance *α*, the 1 − *α* confidence interval will not include the null hypothesis value. Vice versa, if the 1 − *α* confidence interval does not include the null hypothesis value, the *p*-value will be smaller than *α*.

In conclusion, as long as random variability is involved, no effect can be strictly proven merely based on a (small) sample of observations alone. Scientific conclusions must not merely be based on whether a *p*-value passes a user-set threshold without any other supporting evidence or reasoning. Moreover, also a true but possibly small difference might be of no essential practical importance. In general, it is necessary that researchers are aware of what statistical significance testing really means in order not to misuse it. Merely replacing the *p*-value with other methods will not solve the problem, but rather only shift it (Verhulst, 2016; Lakens, 2021). Particularly, completely abolishing any assessment of statistical significance poses the risk of researchers being fooled by random chance.

## 7 Methods

### 7.1 2-CLASTA

#### 7.1.1 Simulations

Simulations were performed as described previously (Arnold et al., 2020). In short, the underlying distribution of biomolecules was simulated on a region of interest of 10 × 10*μ*m^2^. For the simulation of dimers, two biomolecules were assigned to each dimer position. Subsequently, two different types of labels were assigned randomly and competitively to the simulated molecules according to the specified label ratio. For simulation of blinking, a random number of detections was assigned to each label according to blinking statistics determined previously for SNAP-AF647 and SNAP-AF488 (Arnold et al., 2020). Next, the localization coordinates were displaced by random localization errors, which were distributed normally with mean 0 and standard deviation according to the localization precision of 30 nm. Further, to account for experimental errors we included 5 unspecifically bound labels per *μ*m^2^ in each color channel. In addition, we added a background of 1 and 2 signals per *μ*m^2^ for the red and blue color channel, respectively. Background signals were simulated with blinking statistics obtained previously from unlabeled cells (Arnold et al., 2020). If not mentioned otherwise, we used the following parameters: 75 molecules per *μ*m^2^, 40*%* degree of labeling and 1:1 label ratio. All simulations were carried out in MATLAB (R2019b, The MathWorks Inc., Natick, MA) on a standard personal computer.

#### 7.1.2 Calculation of *p*-Value for Multiple Experiment

The overall *p*-value p* for multiple experiments was calculated as $p^{*}=1-\sum_{i=1}^{k-1}\left(\begin{array}{c} m\\ i \end{array}\right)p_{0}^{i}{\left(1-p_{0}\right)}^{m-i}$ , where *m* is the number of performed experiments, *k* the number of observed *p*-values smaller or equal to the threshold *p*_0_, and $\left(\begin{array}{c} m\\ i \end{array}\right)$ denotes the Binomial coefficient. If not stated otherwise, the level of significance for the joint analysis of *p*-values was set to α* = 0.05. As input for the calculation we used the *p*-values derived in (Arnold et al., 2020).

### 7.2 Single Particle Trajectories

#### 7.2.1 Simulation of FRET Trajectories

Simulations were performed as described previously (Schrangl et al., 2018). In short, we first simulated a ground truth state transition trajectory. Here, a two-state model was simulated, characterized by the FRET efficiencies *E*_1_ = 0.2 and *E*_2_ = 0.8 for the two states. Stochastic transitions between the two states were simulated based on the lifetimes *τ*_1_ = 2 and *τ*_2_ = 4 for state 1 and 2, respectively. Subsequently, the state transition trajectory was sampled with finite time resolution according to the exposure time *t*_ex_ = 0.1 and a delay time *t*_del_ = 0.2. All times are given in arbitrary units. For simulation of the fluorescence signal, the donor brightness *d*(*t*_*i*_) and acceptor brightness *a*(*t*_*i*_) at each time point *t*_*i*_ were randomly drawn from a lognormal distribution with mean values *m*_don_(*t*_*i*_) = *n*_phot_(1 − *e*(*t*_*i*_)) and *m*_acc_(*t*_*i*_) = *n*_phot_ *e*(*t*_*i*_), respectively, where *e*(*t*_*i*_) = (*∑*_*i*_ *t*_*i*_ *E*_*i*_)/*t*_ex_ denotes the apparent FRET efficiency and *n*_phot_ = 200 the average number of emitted photons (sum of donor and acceptor fluorophores). The standard deviation *σ* of the lognormal distribution was calculated via *σ* = 0.3 m − 13.61 for the donor, and *σ* = 0.3 m − 1.92 for the acceptor, following values determined previously for the fluorophores AF555 and AF647 (Schrangl et al., 2018). The final FRET efficiency was calculated as $E=\frac{a}{d+a}$ . For each simulation run, we simulated 100 trajectories for each group. The lengths of the trajectories was distributed randomly according to a lognormal distribution with a mean of 27.1 and a standard deviation of 35.5. All simulations were carried out in Python on a standard personal computer.

#### 7.2.2 Permutation Test

We compared two groups *A* and *B* and assessed whether they originated from the same distribution, i.e. the null hypothesis. First, the summary statistics *s*_0_ was calculated for the original samples *A*_0_ and *B*_0_ with sample size *n*_*A*_ and *n*_*B*_, respectively. As a summary statistics, we used the difference between the mean of the two samples, i.e. $s_{0}=\mu_{A_{0}}-\mu_{B_{0}}$ . Second, the data from the two samples was pooled to form the set *M*≔*A* ∪ *B*. Next, permutations of the data were created, i.e. the set *M* was split into new samples *A*_*i*_ and *B*_*i*_. For the standard permutation test, all data points were assigned randomly to one of the two groups. The size of the new samples was *n*_*A*_ and *n*_*B*_, respectively. For the block permutation test, data from individual trajectories were kept together, but each trajectory was randomly assigned to one of the new groups *A*_*i*_ or *B*_*i*_. Both groups contained 100 trajectories. For each random permutation *i* = 1, …, 1000 of the data, the summary statistics $s_{i}=\mu_{A_{i}}-\mu_{B_{i}}$ was calculated. Finally, The two sided *p*-value was calculated as the proportion of generated permutations for which the absolute difference |*s*_*i*_| was greater than the value |*s*_0_| observed for the original data.
